# Are inflammatory bowel diseases associated with an increased risk of COVID-19 susceptibility and severity? A two-sample Mendelian randomization study

**DOI:** 10.3389/fgene.2023.1095050

**Published:** 2023-04-21

**Authors:** Qixiong Ai, Bo Yang

**Affiliations:** Department of Gastroenterology and Hepatology, Guizhou Aerospace Hospital, Zunyi, Guizhou, China

**Keywords:** inflammatory bowel disease, ulcerative colitis, Crohn ‘s disease, COVID-19, Mendelian randomization

## Abstract

**Background:** Due to inconsistent findings in observational studies regarding the relationship between inflammatory bowel disease (IBD), encompassing ulcerative colitis (UC) and Crohn’s disease (CD), and COVID-19, our objective is to explore a potential causative correlation between IBD and COVID-19 susceptibility and its severity using a two-sample Mendelian randomization (MR) analysis.

**Methods:** Using summary data from genome-wide association studies, IBD, including UC and CD, were used as exposure instruments, while COVID-19 susceptibility, hospitalization, and very severe illness were employed as the outcome. The five analysis methods were adopted to evaluate the causal relationship between two diseases, with the inverse variance weighted (IVW) method being the most important. Also, sensitivity analyses were done to make sure that the main results of the MR analyses were reliable.

**Results:** In the analysis using five methods, all *p*-values were higher than 0.05. There was no association between IBD and COVID-19 susceptibility, hospitalization, and severity in our MR study. The random-effect model was applied due to the existence of heterogeneity. MR-Egger regression revealed no indication of directional pleiotropy, and sensitivity analysis revealed similar relationships.

**Conclusion:** This MR study found no evidence to support that IBD (which includes UC and CD) increases the risk of COVID-19 susceptibility or severity. Our result needs further confirmation through larger epidemiological studies.

## Introduction

Coronavirus disease 2019 (COVID-19) is a contagious illness that has spread throughout the world and primarily affects the respiratory system (Guan et al., 2020). By 28 October 2022, a total of 626,337,158 patients had been diagnosed, with 6,566,610 confirmed deaths globally (https://covid.who.int/). Until now, the virus has expanded at an increasing rate, and the pandemic has swiftly spread to many nations. Global nations are bearing a significant socioeconomic burden as a result of the isolation and treatment measures implemented in response to COVID-19. As a result, one of the most important ways to prevent COVID-19 right now is to find the likely risk factors for the disease and take preventive measures for people who are at high risk.

Several studies have shown that COVID-19 susceptible risk factors include white blood cells, type 2 diabetes, obesity, and smoking (Leong et al., 2021; Sun et al., 2021; Au Yeung et al., 2022; Cao et al., 2022). Inflammatory bowel disease (IBD) is a group of chronic, non-specific inflammatory diseases affecting the gut, for which the cause is still unknown. The two main types of IBD are Crohn’s disease (CD) and ulcerative colitis (UC) (Ananthakrishnan, 2015). There may be an increased risk of infection in IBD patients due to immune system imbalance and prolonged use of immunosuppressive drugs, and COVID-19-related symptoms could potentially exacerbate inflammation in the intestines. (Geremia et al., 2014). According to recent research, immune-mediated IBD may enhance the risk of COVID-19 infection (Derikx et al., 2021). Likewise, additional research revealed that age exceeding 65 years and active IBD were among the factors that correlated with heightened susceptibility to COVID-19. (Bezzio et al., 2020). This might be due to the abnormal intestinal immune response, the infiltration of neutrophils, lymphocytes, and plasma cells into the intestinal mucosa, and the disorder of cytokine secretion that occurs during IBD activity (Cassinotti et al., 2014). On the other hand, contrary studies suggest that IBD patients do not have a higher rate of COVID-19 infection compared to the general population. (Macaluso and Orlando, 2020; Monteleone and Ardizzone, 2020; Popa et al., 2020). Non-etheless, these results are susceptible to confounding variables and reverse causation, which cannot be completely ruled out in observational research. Further investigation is required to identify the link between IBD and the COVID-19 infection and severity.

Mendelian randomization (MR) is based on the assumption that genetic variations are randomly distributed in the population and not associated with confounding factors. It uses genetic variants as instrumental variables (IVs) to explore causality between exposure and outcome. (Burgess et al., 2020). MR is based on the random distribution of gametes during meiosis, which allows it to circumvent the confusion and reverse causation that frequently plague observational studies. (Holmes et al., 2017). The goal of this research was to explore whether there was a link between IBD (including UC and CD) and COVID-19 susceptibility and severity using a two-sample MR analysis.

## Methods

### Study design

**FIGURE 1:**
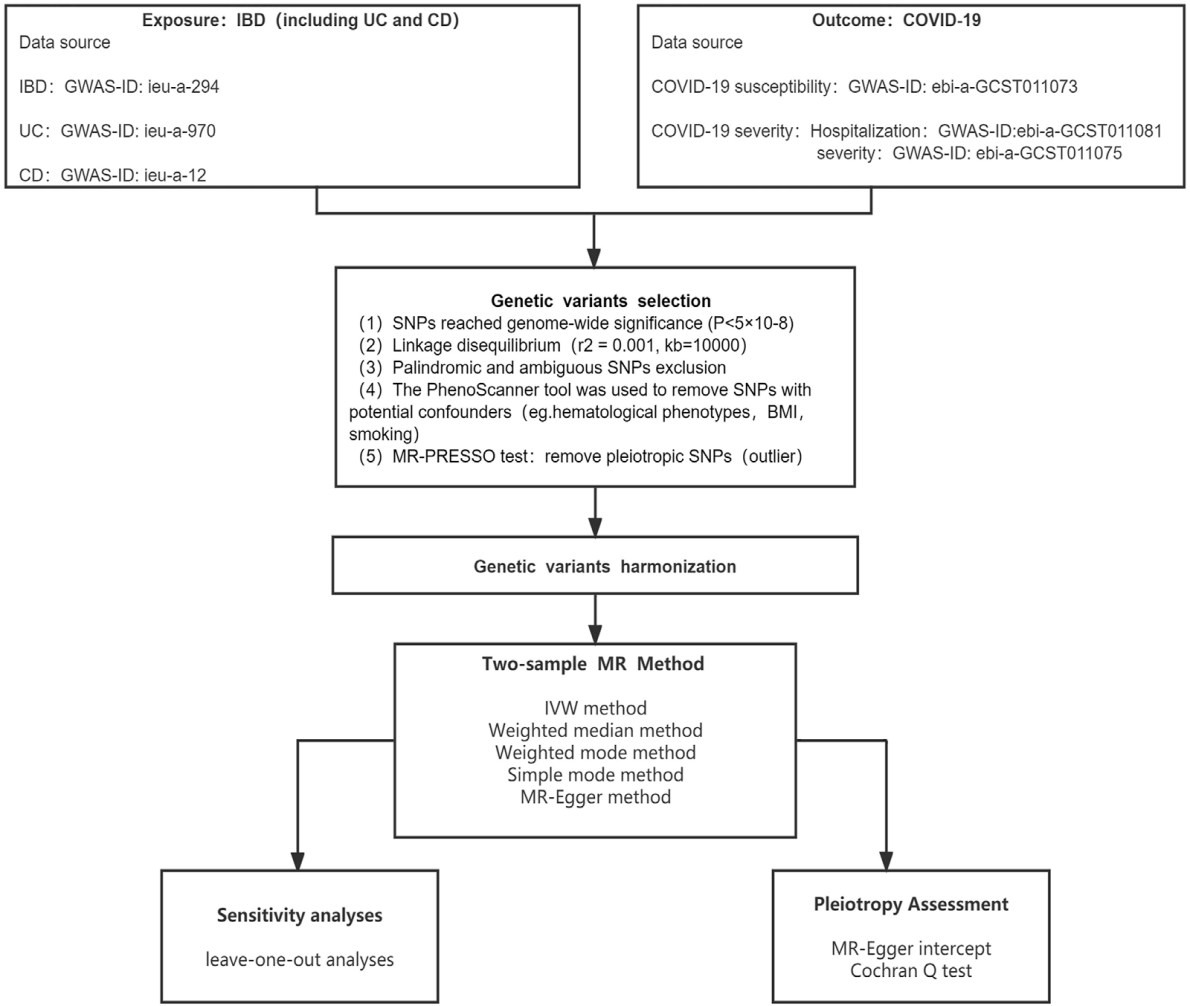
The Mendelian randomization (MR) analysis pipeline of the current study.

The whole research plan is shown in Figure 1. Specifically, the MR method consists of two primary steps: First, randomizing participants based on IVs; then, evaluating the causal relationships between IBD and COVID-19 outcomes (Davey Smith and Hemani, 2014; Emdin et al., 2017). The IVs must adhere to three essential criteria: 1) that IVs and IBD are tightly associated; 2) that IVs and confounders are unrelated; and 3) that IVs should only impact COVID-19 results via IBD, not through other routes (Davies et al., 2018).

### Data source

An important part of running MR analysis was choosing relevant genetic variants. The IEU GWAS database offers users the opportunity to get GWAS summary statistics. The database contains a large number of genetic variants from GWAS summary-level datasets for search or download (Hemani et al., 2018). Consequently, we selected SNPs as IVs for exposures and outcomes using this database. All SNPs and associated summary data were acquired from studies involving solely European populations to mitigate the effects of population stratification. From the Inflammatory Bowel Disease Genetics Consortium, genetic variations linked to IBD were extracted (Liu et al., 2015). IBD was diagnosed using imaging, endoscopic, and histological examinations. We chose SNPs as IVs for IBD, UC, and CD (GWAS ID: ieu-a-294; ieu-a-970; ieu-a-12). From the COVID-19 Host Genetics Initiative round 5, the COVID-19 susceptibility and severity analysis data were collected. We chose SNPs as IVs for COVID-19 susceptibility, hospitalization, and severity (GWAS-ID: ebia-GCST011073; ebia-GCST011081; ebia-GCST011075). Supplementary Table S1 displays detailed characteristics.

All of the information was taken from previously published GWAS summary data that was made accessible to the public. As a result, neither ethical approval nor patient consent were required for the research.

### Selection of instrumental variables

The SNPs that were eligible were selected using a variety of quality control techniques. Appropriate SNPs utilized as IVs must be strongly linked to IBD (p < 5E−08). A clumping algorithm (*r* ^2^ = 0.001, kb = 10,000) was performed to confirm the independence of SNPs and eliminate linkage disequilibrium (LD). The PhenoScanner database was used to filter out the identified SNPs that were linked to other phenotypes and potentially influencing results. When COVID-19 was identified as the outcome, hematological phenotypes (e.g., platelet count, percentage of neutrophils in granulocytes, and lymphocyte count), type 2 diabetes, obesity, and smoking were identified as confounding variables (Leong et al., 2021; Sun et al., 2021; Au Yeung et al., 2022; Cao et al., 2022). To further evaluate the instrumental value of SNPs, we computed F-statistics, with an F-statistic of more than 10 considered reliable. In addition, the MR-PRESSO test was used to verify whether pleiotropy existed and to manually delete outlier SNPs (*p* < 0.05). After eliminating these outlier SNPs, the remaining SNPs were used for subsequent MR analysis.

### Mendelian randomization analysis

The effects of IBD on COVID-19 susceptibility and severity were explored using a variety of methods, the most important of which were the inverse variance weighted (IVW), followed by the Mendelian randomization-Egger (MR-Egger), the weighted median, the weighted mode, and the simple mode. The IVW method has the best statistical validity and reliably calculates the causal impact of exposure on the outcome (Burgess et al., 2013). Pleiotropy, heterogeneity, and sensitivity analyses were used to check the quality. The Cochran’s Q test and the MR-Egger intercept test were employed to investigate heterogeneity and directed horizontal pleiotropy, respectively, to confirm the reliability of the findings. If there was no evidence of heterogeneity, the fixed-effect model was employed; otherwise, the random-effect model was used. Additionally, we evaluated the consistency and effectiveness of MR findings using the “leave-one-out” method.

### Statistical analysis

The statistical analyses were carried out in R version 4.1.3 using the “TwoSampleMR” and “MRPRESSO” packages, respectively (Hemani et al., 2018; Verbanck et al., 2018). There was no heterogeneity across IVs when the Q statistic was *p* > 0.05, but there was heterogeneity when *p* < 0.05. If the MR-Egger regression intercept was not zero and *p* < 0.05, the IV was thought to exhibit horizontal pleiotropy. On the other hand, if *p* > 0.05, the results were considered not to have horizontal pleiotropy. Regarding the correction for multiple testing, we employed a Bonferroni correction to reduce the likelihood of type 1 error, thereby improving the reliability of our results. The Bonferroni-correction (0.0055, 0.05/3 exposures/3 outcomes) was employed to account for the issue of multiple testing. A possible correlation was considered to exist when the *p*-value was less than 0.0055.

## Results

### Filter instrument variables

After applying stringent exclusion criteria, we included 134, 88, and 122 SNPs as IVs for IBD, UC, and CD, respectively. We found and removed 9 (IBD), 4 (UC), and 7 (CD) palindromic SNPs and 13 (IBD), 10 (UC), and 16 (CD) ambiguous SNPs. Using PhenoScanner, 46 (IBD), 33 (UC), and 52 (CD) SNPs were manually removed. Two SNPs (rs2143178 and rs516246) in IBD, one SNP (rs9611131) in UC, and two SNPs (rs2413583 and rs516246) in CD were excluded based on the MR-PRESSO test. Finally, after strict screening, 66 SNPs (IBD), 40 SNPs (UC), and 45 SNPs (CD) qualified as IVs for the MR analysis. The F-statistics of all three IVs were more than 10 (ranging from 100.2550 to 3614.1141 for IBD; 110.0008 to 1571.1295 for UC; and 114.3503 to 3044.4854 for CD). The characteristics of SNPs for IBD are shown in Supplementary Tables S2–S4.

### Association between IBD and COVID-19 susceptibility and severity

**FIGURE 2:**
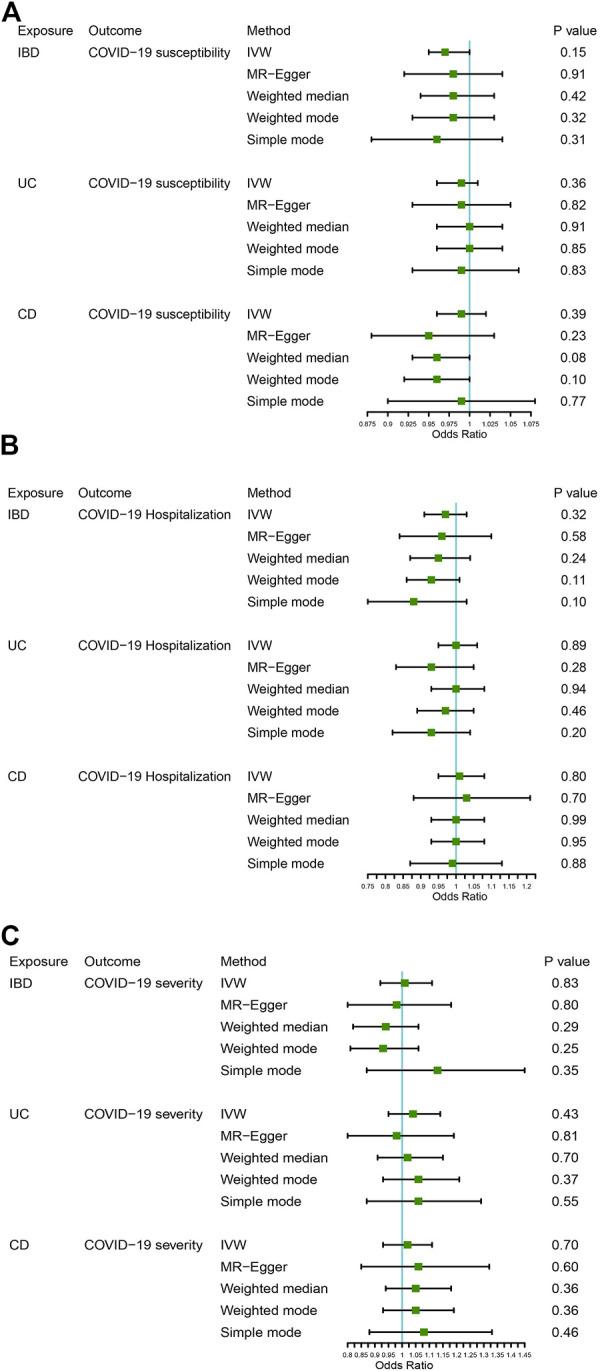
The association between IBD (including UC and CD) and COVID-19 susceptibility, hospitalization, and severity. **(A)** COVID-19 susceptibility, **(B)** COVID-19 hospitalization, **(C)** COVID-19 severity.

**FIGURE 3:**
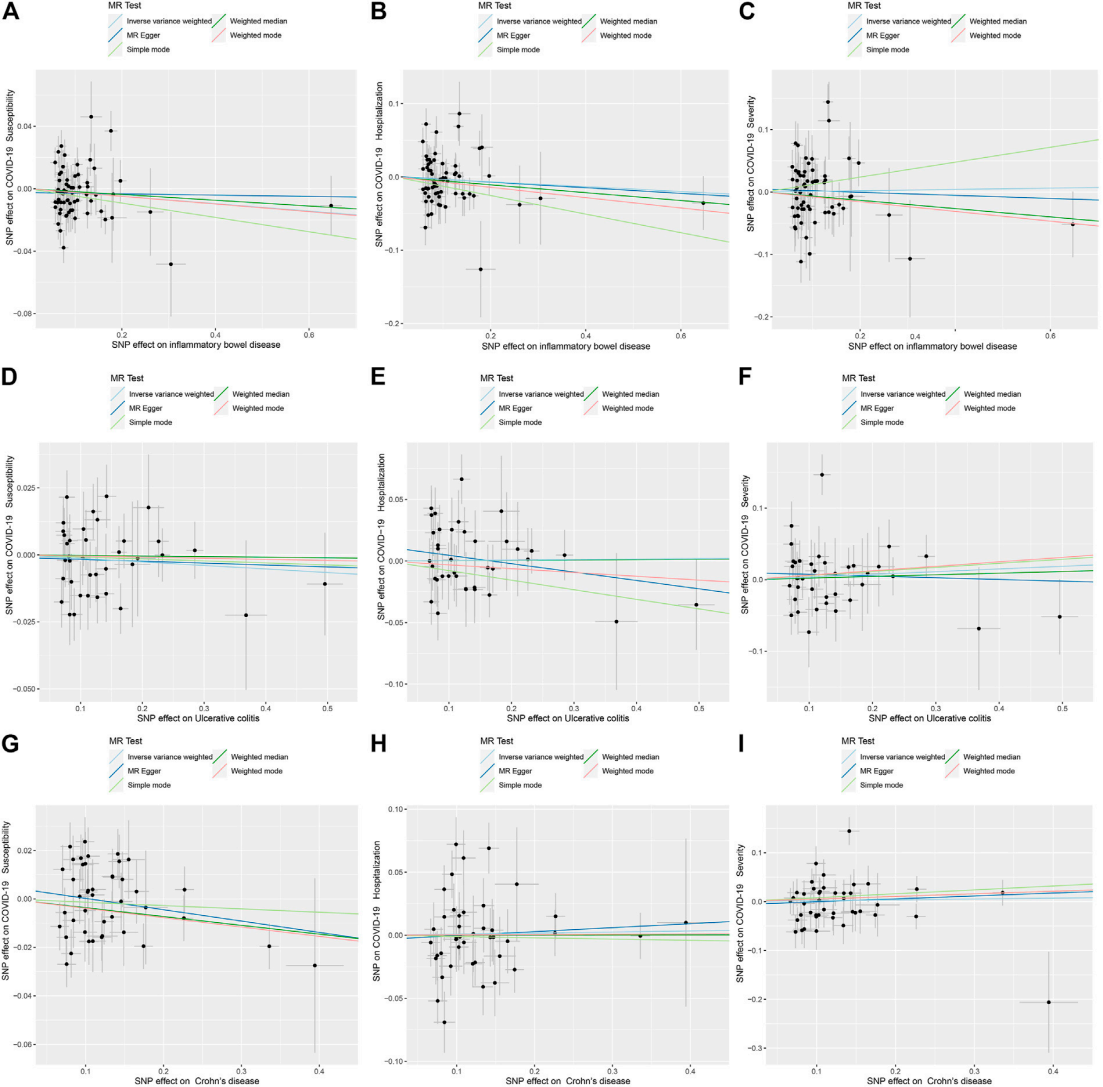
Scatter plots for causal SNP effect of IBD (including UC and CD) on COVID-19 susceptibility, hospitalization, and severity. **(A)** Effect of IBD on COVID-19 susceptibility; **(B)** Effect of IBD on COVID-19 hospitalization; **(C)** Effect of IBD on COVID-19 severity; **(D)** Effect of UC on COVID-19 susceptibility; **(E)** Effect of UC on COVID-19 hospitalization; **(F)** Effect of UC on COVID-19 severity; **(G)** Effect of CD on COVID-19 susceptibility; **(H)** Effect of CD on COVID-19 hospitalization; **(I)** Effect of CD on COVID-19 severity.

Figure 2 displays the results of MR analysis, which demonstrated that none of the five methods had a statistically significant relationship with IBD (including UC and CD) and COVID-19 susceptibility, hospitalization or severity (all *p* > 0.0055). The sensitivity studies, such as the Cochran’s Q test and the MR-Egger intercept test, were performed to evaluate the robustness of the aforementioned findings (Table 1). The scatter plots of association estimates between IBD (including UC and CD) and COVID-19 susceptibility, hospitalization, and severity, as well as the MR causal estimates, are shown in Figure 3. The Cochran’s Q test, however, revealed heterogeneity between IBD and COVID-19 susceptibility, hospitalization, or severity. As a result, the random-effect model was used in the IVW approach (heterogeneity *p*-value<0.05). Using the MR-Egger intercept test, it was discovered that there was no existence of directional pleiotropy (Intercept *p*-value>0.05). The consistency of the MR impact estimates was further confirmed by the leave-one-out method (Supplementary Figure S1). Forest plots of MR analysis of IBD (including UC and CD) and COVID-19 susceptibility, hospitalization, and severity are displayed in Supplementary Figure S2. The funnel plots revealed the heterogeneity in the estimations for each SNP (Supplementary Figure S3).

**TABLE 1:** Heterogeneity and horizontal pleiotropy analyses results.

Exposure	Outcome	Cochran Q statistic (IVW method)	Heterogeneity *p*-value (IVW method)	MR-Egger	
				Intercept	Intercept *p*-value
IBD	COVID-19 susceptibility	105.9348	0.0005	−0.0024	0.5198
	COVID-19 hospitalization	113.4307	1.02E-04	0.0006	0.9319
	COVID-19 severity	108.1956	0.0003	0.0045	0.6856
UC	COVID-19 susceptibility	43.5409	0.2129	−0.0009	0.8239
	COVID-19 hospitalization	49.2361	0.0859	0.0114	0.1943
	COVID-19 severity	58.1414	0.0147	0.0099	0.4887
CD	COVID-19 susceptibility	71.1531	0.0032	0.0048	0.3533
	COVID-19 hospitalization	80.0273	0.0003	−0.0034	0.7546
	COVID-19 severity	71.8791	0.0027	−0.0062	0.6819

## Discussion

This work used large-scale GWAS data from the IBDGC and the COVID-19 Host Genetics Initiative Round 5 to assess the probable causal connection of IBD with COVID-19 susceptibility and severity. This is the first MR study, to our knowledge, to explore the causal relationship between IBD and COVID-19 risk. The MR analysis revealed that there was insufficient evidence to suggest that IBD (UC and CD) may enhance COVID-19 susceptibility, hospitalization, and severity.

In terms of clinical presentation, IBD and COVID-19 share similar symptoms, such as stomach discomfort, diarrhea, pneumonia, and so on. In a prior study of 709 IBD patients, 53 were shown to be concurrently infected with COVID-19 (Vigano et al., 2020). The researchers found that the proportion of people with IBD and COVID-19 who also suffered from diarrhea was 49%, which was a significant increase above the proportion of people with only IBD. An earlier observational study revealed that patients with IBD are more likely to infect COVID-19, particularly when experiencing active disease or taking immunosuppressive therapy (Bezzio et al., 2020). Another study found that SARS-CoV2, which is widely expressed in the lung and gut, has been shown to be an inflammatory protective factor that is downregulated and upregulated, respectively, in COVID-19 and IBD, suggesting the presence of a coregulatory mechanism (Tao et al., 2022). However, there are no published studies that detail the underlying process. In line with the majority of other studies, our MR analysis showed no robust evidence of a connection between IBD and COVID-19 susceptibility, hospitalization, or severity. A large countrywide cohort study in the Netherlands compared the incidence of COVID-19 in people with IBD to that in the general population and found no statistically significant difference (Derikx et al., 2021). Likewise, the risk of COVID-19 infection was also shown to be the same in both the IBD and non-IBD groups, according to a multi-center network investigation (Singh et al., 2020). In addition, a recent meta-analysis and comprehensive review also showed the same result regarding IBD not increasing COVID-19 morbidity and mortality (Lee et al., 2023). Reverse causality confounding and other biases in observational studies may alter the causal effects of illness exposure on outcomes, resulting in incorrect results. The reported causal links between IBD and COVID-19 outcomes may be messed up in observational research, likely because of confounding factors like hematological phenotypes, type 2 diabetes, body mass index (BMI), and smoking. The MR analysis takes advantage of strong genetic variation in order to produce more reliable evidence for predicting the cause of illness (Davey Smith and Hemani, 2014; Bowden and Holmes, 2019). MR is increasingly used to infer causal relationships between exposures and outcomes. It can now be speculated that the data does not support the causal link between IBD and COVID-19 infection and severity, taking into account both the information already available and the findings of our investigation.

There is no question that our research has certain shortcomings as well. Firstly, since this research only included people of European heritage, the findings cannot be applied to other ethnic groups. Therefore, future MR studies in non-European populations would be valuable to further confirm the causal relationship between IBD and COVID-19 infection and severity. Secondly, genetic instruments may impact outcomes via other confounding variables. Tight exclusion criteria and the PhenoScanner tool can exclude genetic instruments related to confounding factors as much as possible, but this cannot be totally eliminated. Thirdly, in the MR model, we only included a linear impact association between IBD and COVID-19. Additionally, we were unable to investigate the non-linearity of the link between IBD and COVID-19 using the GWAS summary data. Fourth, we failed to perform a stratified analysis based on active or remission periods in IBD due to the limited available datasets. Fifth, as the infectiousness of COVID-19 is a dynamic outcome that is influenced by other confounding factors such as social restrictions, the interpretation and understanding of the study results are more challenging and complex. Hence, considering these limits, evaluating patient hospitalization and severity at the same time may provide more persuasive and reliable results, which can help to comprehensively understand the relationship between IBD and COVID-19. Finally, we discovered that COVID-19 infection risk was not directly influenced by IBD, although the underlying molecular mechanism was still unknown. It was necessary to conduct further functional experiments to verify our conclusions.

## Conclusion

Overall, the cause-and-effect connection between IBD and COVID-19 susceptibility and severity were assessed using the two-sample MR method. Based on the findings of this MR investigation, it seems that IBD does not appear to increase the risk of COVID-19 susceptibility, hospitalization, and severity. To validate our findings, we need to do more large-scale epidemiological studies and more research into the biological link between IBD and COVID-19.

## Data Availability

The original contributions presented in the study are included in the article/Supplementary Material, further inquiries can be directed to the corresponding author.
