# Supplementary material for: Are inflammatory bowel diseases associated with an increased risk of COVID-19 susceptibility and severity? A two-sample Mendelian randomization study
Source: Front Genet. 2023 Apr 21;14:1095050. doi: 10.3389/fgene.2023.1095050 (PMC10160392; doi:10.3389/fgene.2023.1095050)
Supplement: Supplementary file 1 [file Table1.DOCX]

Supplementary Material

## Supplementary Figures

**Supplementary Figure 1. “Leave-one-out” sensitivity analysis of the causal effects of inflammatory bowel disease (including UC and CD) on COVID-19 susceptibility, hospitalization, and severity.**


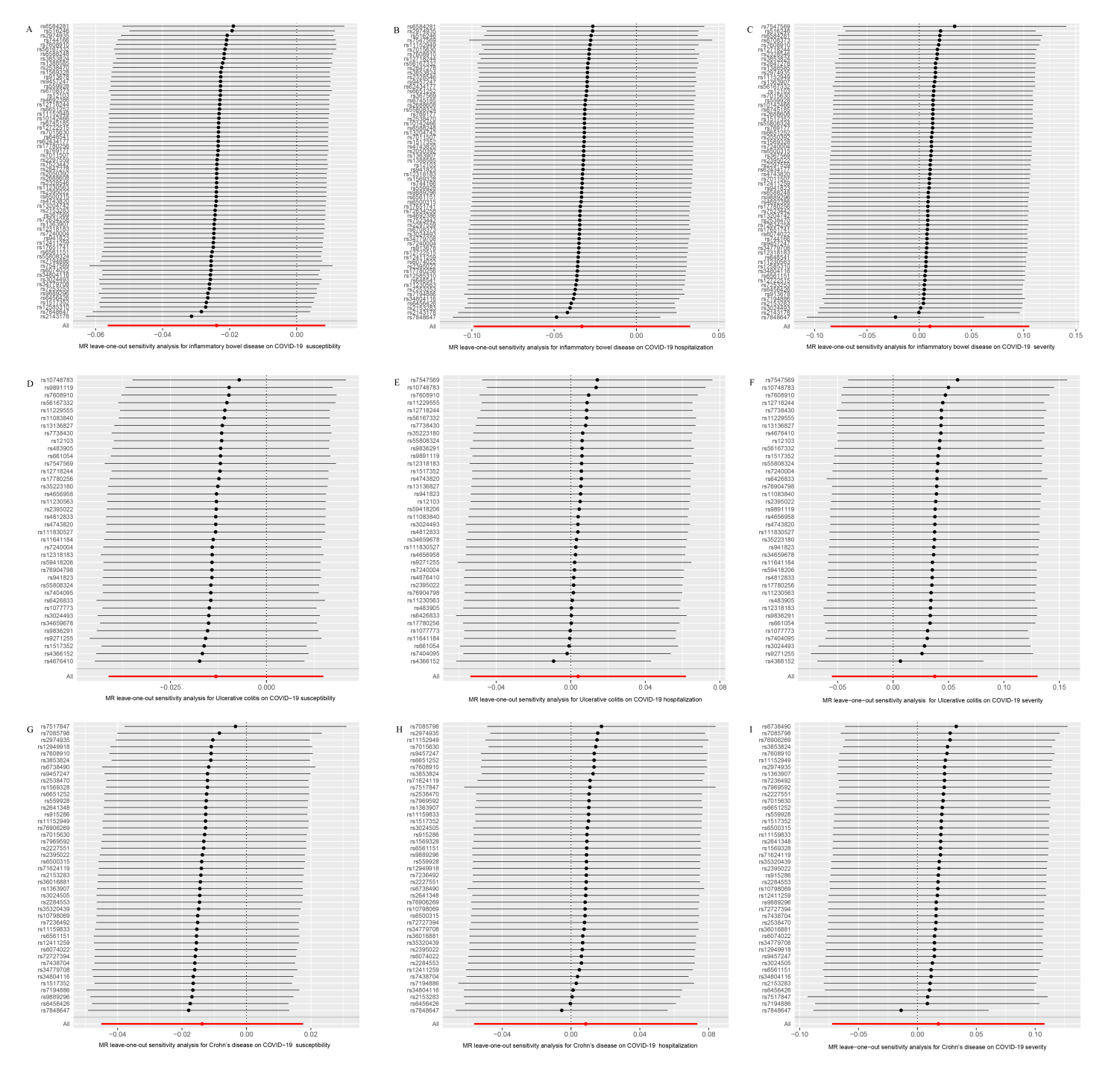


Note: (A) Effect of IBD on COVID-19 susceptibility; (B) Effect of IBD on COVID-19 hospitalization; (C) Effect of IBD on COVID-19 severity; (D) Effect of UC on COVID-19 susceptibility; (E) Effect of UC on COVID-19 hospitalization; (F) Effect of UC on COVID-19 severity; (G) Effect of CD on COVID-19 susceptibility; (H) Effect of CD on COVID-19 hospitalization; (I) Effect of CD on COVID-19 severity

**Supplementary Figure 2. Forest plot of the Mendelian randomization (MR) outcome.**


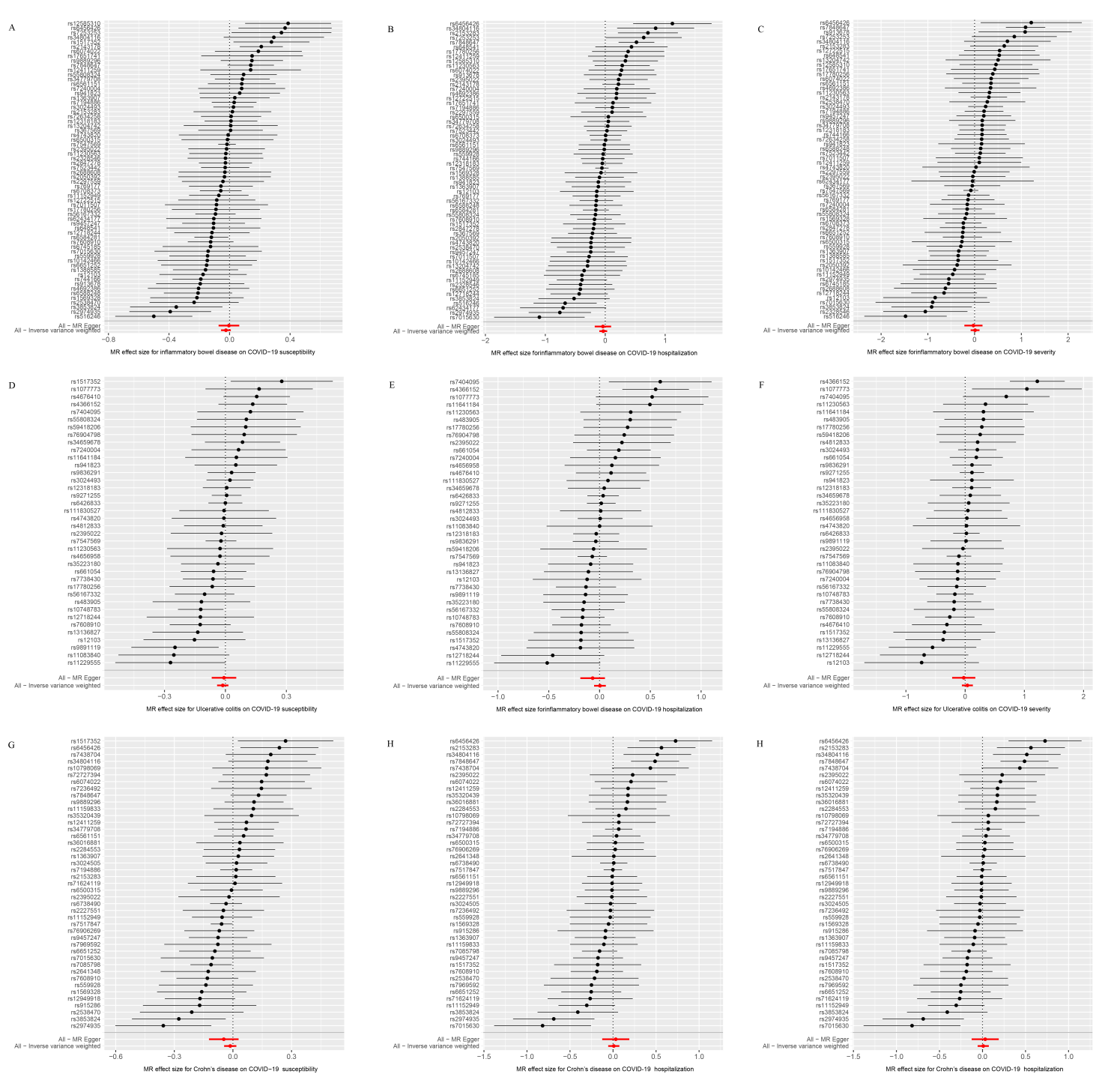


Note: (A) Effect of IBD on COVID-19 susceptibility; (B) Effect of IBD on COVID-19 hospitalization; (C) Effect of IBD on COVID-19 severity; (D) Effect of UC on COVID-19 susceptibility; (E) Effect of UC on COVID-19 hospitalization; (F) Effect of UC on COVID-19 severity; (G) Effect of CD on COVID-19 susceptibility; (H) Effect of CD on COVID-19 hospitalization; (I) Effect of CD on COVID-19 severity

**Supplementary Figure 3. Funnel plot of genetic associations with exposure against causal estimates based on each genetic variant individual.**

**
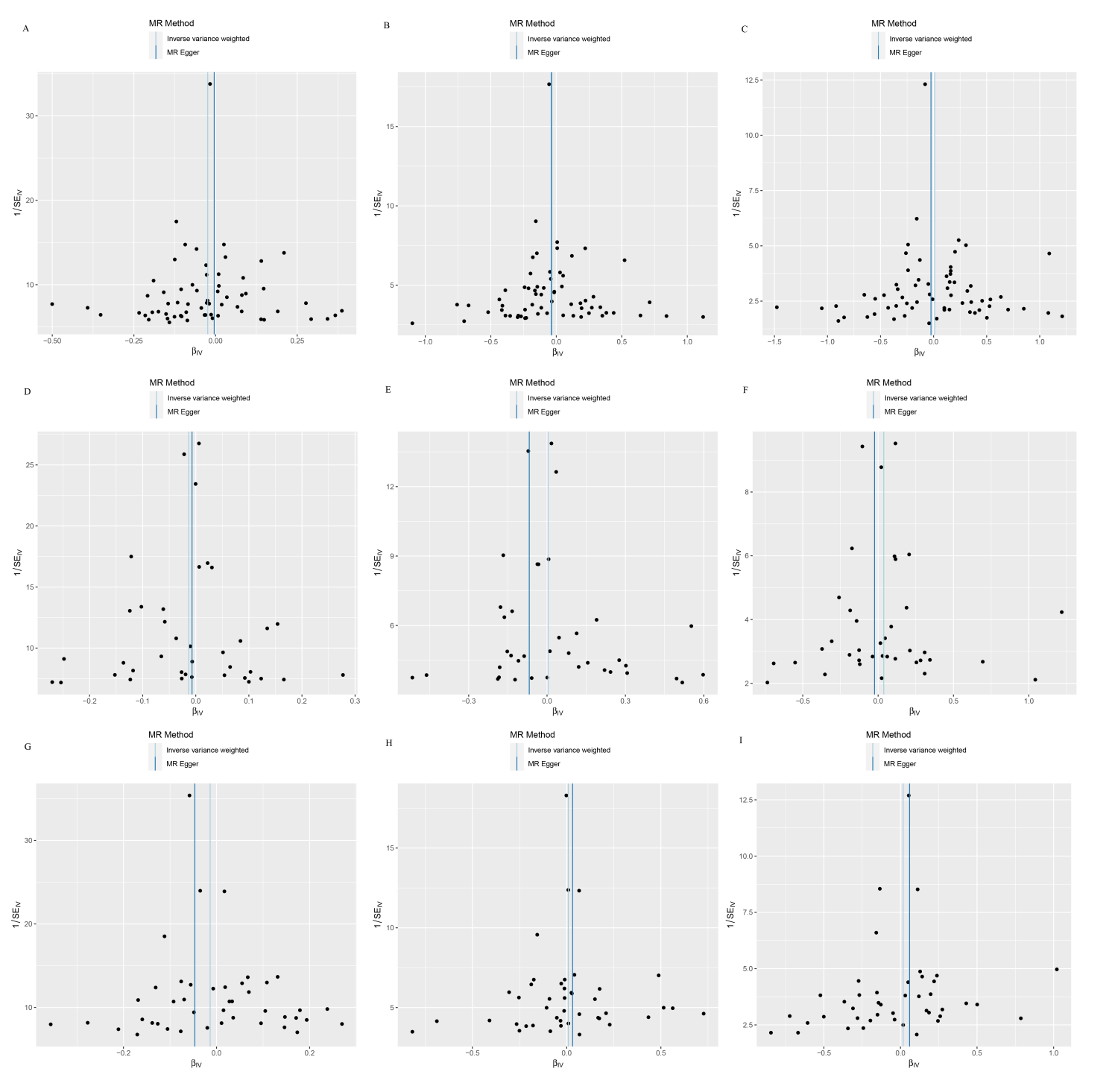
**

Note: (A) Effect of IBD on COVID-19 susceptibility; (B) Effect of IBD on COVID-19 hospitalization; (C) Effect of IBD on COVID-19 severity; (D) Effect of UC on COVID-19 susceptibility; (E) Effect of UC on COVID-19 hospitalization; (F) Effect of UC on COVID-19 severity; (G) Effect of CD on COVID-19 susceptibility; (H) Effect of CD on COVID-19 hospitalization; (I) Effect of CD on COVID-19 severity

**Supplementary Table.**

**Supplementary Table 1. Sources of data for the analysis**

| Phenotype | Source of genetic variants | |
| --- | --- | --- |
|  | Consortium | Participants |
| Exposure |  |  |
| IBD | IBDGC | Cases: 31,665  Controls:33,977  Number of SNPs:157,116 |
| UC | IBDGC | Cases: 13,768  Controls:33,977  Number of SNPs:156,116 |
| CD | IBDGC | Cases: 13,768  Controls:33,977  Number of SNPs:156,116 |
| Outcome |  |  |
| COVID-19 Susceptibility | COVID-19 Host Genetics Initiative round 5 | Cases: 38,984  Controls:1,644,784  Number of SNPs:8,660,177 |
| COVID-19 Hospitalization | COVID-19 Host Genetics Initiative round 5 | Cases: 9,986  Controls:1,877,672  Number of SNPs:8,107,040 |
| COVID-19  Severity | COVID-19 Host Genetics Initiative round 5 | Cases: 5,101  Controls:1,383,241  Number of SNPs:9,739,225 |

Abbreviations: ulcerative colitis (UC); Crohn's disease (CD)

**Supplementary Table 2. Characteristics of IBD-associated SNPs.**

| SNP | Pos | P | Beta | Chr | Samplesize | SE | EA | NEA | EAF | F |
| --- | --- | --- | --- | --- | --- | --- | --- | --- | --- | --- |
| rs7523442 | 20165971 | 2.76439E-36 | 0.124537 | 1 | 65642 | 0.00990061 | T | C | 0.5356 | 510.3779514 |
| rs72634258 | 8150638 | 1.24652E-19 | -0.126877 | 1 | 65642 | 0.0139965 | C | T | 0.1757 | 307.5043222 |
| rs2974935 | 155181843 | 8.86748E-12 | 0.0687256 | 1 | 65642 | 0.0100715 | T | G | 0.4948 | 155.365796 |
| rs12411259 | 172866210 | 6.17504E-09 | 0.0669054 | 1 | 65642 | 0.0115117 | A | G | 0.2401 | 107.3938683 |
| rs3024493 | 206943968 | 1.64778E-50 | 0.196922 | 1 | 65642 | 0.0131753 | A | C | 0.1572 | 681.4746644 |
| rs12103 | 1247494 | 3.2802E-11 | -0.0867354 | 1 | 65642 | 0.0130755 | C | T | 0.8166 | 148.2449044 |
| rs7547569 | 67731368 | 1.652E-170 | -0.6472 | 1 | 65642 | 0.0232516 | C | T | 0.06675 | 3614.114102 |
| rs2297559 | 160854526 | 1.88495E-11 | 0.0741659 | 1 | 65642 | 0.0110454 | A | G | 0.6822 | 156.9313771 |
| rs6588248 | 67652984 | 1.38484E-16 | 0.0819867 | 1 | 65642 | 0.0099185 | G | T | 0.5297 | 220.5704085 |
| rs1517352 | 191931464 | 3.86545E-14 | 0.0778816 | 2 | 65642 | 0.0102944 | C | A | 0.6048 | 190.8789767 |
| rs6708373 | 234172846 | 1.42561E-41 | 0.134178 | 2 | 65642 | 0.0099341 | G | A | 0.5277 | 594.4034106 |
| rs7608910 | 61204856 | 2.59956E-36 | 0.126444 | 2 | 65642 | 0.0100484 | G | A | 0.3909 | 503.5799317 |
| rs6745185 | 241586960 | 1.37322E-09 | 0.0698267 | 2 | 65642 | 0.0115253 | G | T | 0.7389 | 123.7234336 |
| rs4692386 | 26132361 | 1.21194E-08 | 0.0579752 | 4 | 65642 | 0.0101746 | C | T | 0.593 | 106.6687885 |
| rs7711427 | 40414886 | 4.62914E-66 | 0.174764 | 5 | 65642 | 0.0101797 | C | A | 0.613 | 965.1914559 |
| rs34804116 | 72539850 | 3.62402E-08 | -0.0574631 | 5 | 65642 | 0.0104322 | A | C | 0.3867 | 102.9685378 |
| rs1363907 | 96252803 | 4.86519E-15 | 0.0815026 | 5 | 65642 | 0.0104086 | A | G | 0.4212 | 213.2884184 |
| rs56167332 | 158827769 | 7.16638E-50 | 0.155855 | 5 | 65642 | 0.0104967 | A | C | 0.3375 | 720.8469322 |
| rs6456426 | 21438889 | 8.18276E-11 | -0.0643405 | 6 | 65642 | 0.0099028 | A | C | 0.4984 | 136.1453584 |
| rs13204742 | 128245765 | 5.39312E-10 | 0.0916208 | 6 | 65642 | 0.0147604 | T | G | 0.1267 | 122.1614866 |
| rs769177 | 31547611 | 6.5283E-20 | 0.260903 | 6 | 65642 | 0.0285603 | T | C | 0.02548 | 222.6473368 |
| rs62434177 | 138087506 | 1.13951E-08 | -0.179105 | 6 | 65642 | 0.031375 | A | G | 0.03236 | 132.1325038 |
| rs9457247 | 167392174 | 2.48085E-18 | 0.089151 | 6 | 65642 | 0.0102086 | T | C | 0.5398 | 260.2248847 |
| rs2328546 | 20657345 | 1.29718E-13 | 0.0940162 | 6 | 65642 | 0.0126938 | C | T | 0.8014 | 185.2064875 |
| rs11152949 | 106449085 | 7.2477E-23 | 0.105057 | 6 | 65642 | 0.0106717 | G | A | 0.3195 | 316.5460382 |
| rs2538470 | 148220448 | 2.99916E-11 | -0.0675599 | 7 | 65642 | 0.0101646 | G | A | 0.6378 | 138.715932 |
| rs12718244 | 50175654 | 3.35274E-14 | 0.0761709 | 7 | 65642 | 0.0100437 | A | G | 0.4081 | 184.5060934 |
| rs2395022 | 98750379 | 8.26799E-15 | -0.181635 | 7 | 65642 | 0.0233963 | C | A | 0.95885 | 171.3366597 |
| rs7011507 | 49129242 | 2.03222E-08 | -0.0846011 | 8 | 65642 | 0.0150825 | A | G | 0.1233 | 101.7272276 |
| rs7015630 | 90875918 | 2.89694E-08 | -0.0627799 | 8 | 65642 | 0.0113167 | C | T | 0.2657 | 101.1051594 |
| rs6651252 | 129567181 | 9.08239E-10 | -0.0908484 | 8 | 65642 | 0.014833 | C | T | 0.13 | 122.7742508 |
| rs7848647 | 117569046 | 3.15791E-35 | 0.13239 | 9 | 65642 | 0.0106897 | C | T | 0.6746 | 509.0115115 |
| rs4743820 | 93928416 | 3.79656E-09 | 0.0639523 | 9 | 65642 | 0.0108526 | T | C | 0.7019 | 112.5361515 |
| rs34779708 | 35466185 | 2.073E-25 | 0.106679 | 10 | 65642 | 0.0102408 | G | T | 0.3512 | 342.2000022 |
| rs2050392 | 30691503 | 1.87241E-11 | 0.0691178 | 10 | 65642 | 0.0102921 | A | G | 0.6003 | 150.8264961 |
| rs12722515 | 6081230 | 4.56773E-12 | -0.0989022 | 10 | 65642 | 0.0142955 | A | C | 0.1627 | 175.4034342 |
| rs2153283 | 59972299 | 1.53568E-11 | -0.0859637 | 10 | 65642 | 0.0127458 | A | C | 0.217 | 165.2502924 |
| rs2688608 | 75658349 | 2.75467E-10 | 0.062403 | 10 | 65642 | 0.00988642 | T | G | 0.557 | 126.3874178 |
| rs6584281 | 101286480 | 9.36268E-62 | -0.164639 | 10 | 65642 | 0.00992861 | G | A | 0.5188 | 900.54911 |
| rs11230563 | 60776209 | 1.70608E-14 | -0.081194 | 11 | 65642 | 0.0105845 | T | C | 0.348 | 196.9583823 |
| rs559928 | 64150370 | 3.32583E-13 | 0.094388 | 11 | 65642 | 0.0129645 | C | T | 0.8128 | 178.4434907 |
| rs648541 | 114429934 | 1.21728E-09 | -0.0648616 | 11 | 65642 | 0.0106716 | G | A | 0.3409 | 124.3294522 |
| rs1388585 | 40531691 | 6.8533E-22 | -0.30489 | 12 | 65642 | 0.031707 | A | G | 0.98081 | 230.4980008 |
| rs12318183 | 68503836 | 1.66994E-27 | 0.109531 | 12 | 65642 | 0.01008 | A | C | 0.3854 | 375.1908605 |
| rs941823 | 41013977 | 6.18728E-13 | 0.0830172 | 13 | 65642 | 0.0115361 | C | T | 0.7509 | 169.6726022 |
| rs12585310 | 27528347 | 5.24928E-11 | 0.0706485 | 13 | 65642 | 0.0107636 | A | G | 0.3136 | 141.3487555 |
| rs6561151 | 44484706 | 3.53346E-17 | 0.1 | 13 | 65642 | 0.0118661 | A | G | 0.2235 | 228.6270404 |
| rs1569328 | 75741751 | 3.20959E-09 | -0.0809722 | 14 | 65642 | 0.0136766 | T | C | 0.1702 | 121.7891166 |
| rs10142466 | 69271784 | 1.08365E-08 | -0.0580054 | 14 | 65642 | 0.010146 | G | A | 0.5065 | 110.5944002 |
| rs55808324 | 88444752 | 5.13452E-17 | 0.141213 | 14 | 65642 | 0.016844 | A | G | 0.09318 | 221.9513262 |
| rs17651741 | 38869666 | 2.80892E-08 | 0.0702476 | 15 | 65642 | 0.0126505 | A | G | 0.191 | 100.2550485 |
| rs6500315 | 50508101 | 1.12065E-10 | 0.0766082 | 16 | 65642 | 0.0118778 | G | A | 0.7753 | 134.4964745 |
| rs367569 | 11365500 | 1.92664E-17 | -0.0958166 | 16 | 65642 | 0.011275 | T | C | 0.2891 | 248.6444021 |
| rs7194886 | 50725193 | 2.53338E-36 | -0.126026 | 16 | 65642 | 0.0100135 | T | C | 0.4357 | 516.6799543 |
| rs3853824 | 54880993 | 7.70123E-10 | 0.064031 | 17 | 65642 | 0.0104099 | C | T | 0.6385 | 124.4718385 |
| rs744166 | 40514201 | 1.14446E-22 | -0.100017 | 17 | 65642 | 0.0102076 | G | A | 0.4204 | 321.5582334 |
| rs9889296 | 32570547 | 1.35301E-20 | -0.104999 | 17 | 65642 | 0.0112854 | A | G | 0.2723 | 288.0517911 |
| rs17780256 | 70642923 | 3.19374E-11 | -0.083427 | 17 | 65642 | 0.0125693 | C | A | 0.1927 | 142.4524677 |
| rs7240004 | 46395022 | 1.01436E-10 | -0.0665215 | 18 | 65642 | 0.0102898 | G | A | 0.3795 | 137.0825694 |
| rs2847278 | 12778715 | 8.32722E-28 | -0.144528 | 18 | 65642 | 0.0132236 | T | C | 0.841 | 368.7470809 |
| rs7253253 | 10714058 | 6.18586E-09 | -0.134424 | 19 | 65642 | 0.02313 | T | G | 0.95432 | 103.5755063 |
| rs516246 | 49206172 | 1.14842E-13 | 0.0755599 | 19 | 65642 | 0.0101797 | T | C | 0.4652 | 187.0027229 |
| rs913678 | 48955424 | 5.34687E-11 | -0.0691643 | 20 | 65642 | 0.0105418 | C | T | 0.3293 | 138.9956608 |
| rs6074022 | 44740196 | 8.32339E-11 | -0.0742587 | 20 | 65642 | 0.0114338 | T | C | 0.7497 | 136.126078 |
| rs8127691 | 45614860 | 8.97842E-30 | -0.114259 | 21 | 65642 | 0.0100818 | C | T | 0.6132 | 409.0401656 |
| rs2143178 | 39660829 | 4.79954E-38 | -0.176684 | 22 | 65642 | 0.0137017 | C | T | 0.1658 | 571.7606854 |

**Supplementary Table 3. Characteristics of UC-associated SNPs.**

| SNP | Pos | P | Beta | Chr | Samplesize | SE | EA | NEA | EAF | F |
| --- | --- | --- | --- | --- | --- | --- | --- | --- | --- | --- |
| rs3024493 | 206943968 | 1.41971E-43 | 0.226278 | 1 | 47745 | 0.0163471 | A | C | 0.1572 | 656.6499996 |
| rs111830527 | 22687173 | 5.08745E-11 | -0.192324 | 1 | 47745 | 0.0292804 | A | G | 0.05254 | 176.4655278 |
| rs7547569 | 67731368 | 8.70964E-65 | -0.495701 | 1 | 47745 | 0.0291648 | C | T | 0.06675 | 1507.758472 |
| rs12103 | 1247494 | 9.96369E-10 | -0.0995588 | 1 | 47745 | 0.0162944 | C | T | 0.8166 | 142.1670711 |
| rs35223180 | 8185902 | 1.0392E-15 | -0.141003 | 1 | 47745 | 0.0175766 | T | G | 0.1791 | 280.7557532 |
| rs6426833 | 20171860 | 3.77051E-76 | 0.232387 | 1 | 47745 | 0.0125836 | A | G | 0.536 | 1317.867188 |
| rs4656958 | 160856964 | 2.8167E-09 | 0.0824158 | 1 | 47745 | 0.0138702 | G | A | 0.6821 | 141.052383 |
| rs7608910 | 61204856 | 1.25026E-23 | 0.127099 | 2 | 47745 | 0.0126851 | G | A | 0.3909 | 370.1109822 |
| rs1517352 | 191931464 | 2.09991E-09 | 0.0777839 | 2 | 47745 | 0.0129859 | C | A | 0.6048 | 138.4859654 |
| rs4676410 | 241563739 | 1.85481E-19 | 0.142022 | 2 | 47745 | 0.0157426 | A | G | 0.2038 | 314.5788236 |
| rs9836291 | 49697459 | 8.19785E-38 | 0.170257 | 3 | 47745 | 0.0132458 | A | G | 0.2878 | 574.1615708 |
| rs13136827 | 123171318 | 2.34542E-10 | -0.111814 | 4 | 47745 | 0.0176452 | C | T | 0.1622 | 162.780166 |
| rs56167332 | 158827769 | 7.2711E-27 | 0.141368 | 5 | 47745 | 0.0131737 | A | C | 0.3375 | 430.5269344 |
| rs7711427 | 40414886 | 3.67367E-12 | 0.088938 | 5 | 47745 | 0.0127983 | C | A | 0.613 | 179.8534705 |
| rs7738430 | 31508836 | 3.50913E-27 | 0.367964 | 6 | 47745 | 0.0340763 | C | T | 0.02624 | 332.6454159 |
| rs9271255 | 32580357 | 1.31009E-94 | -0.284959 | 6 | 47745 | 0.013809 | T | C | 0.732 | 1571.129556 |
| rs34659678 | 111888540 | 5.95251E-17 | 0.209947 | 6 | 47745 | 0.0250948 | T | C | 0.05734 | 228.5841801 |
| rs1077773 | 17442679 | 5.96458E-09 | 0.0721282 | 7 | 47745 | 0.012398 | A | G | 0.5238 | 124.2319733 |
| rs12718244 | 50175654 | 1.4115E-08 | 0.0717762 | 7 | 47745 | 0.0126545 | A | G | 0.4081 | 119.1236024 |
| rs2395022 | 98750379 | 2.87919E-10 | -0.183896 | 7 | 47745 | 0.029166 | C | A | 0.95885 | 127.7512967 |
| rs4366152 | 117564875 | 7.79471E-19 | 0.120371 | 9 | 47745 | 0.0135814 | C | T | 0.68 | 302.9629534 |
| rs4743820 | 93928416 | 4.04772E-09 | 0.0809228 | 9 | 47745 | 0.0137572 | T | C | 0.7019 | 131.1929454 |
| rs10748783 | 101285872 | 7.73393E-39 | -0.164756 | 10 | 47745 | 0.0126394 | A | C | 0.5237 | 655.4003022 |
| rs59418206 | 35331624 | 1.45001E-08 | 0.0735513 | 10 | 47745 | 0.012978 | A | G | 0.3508 | 117.9315333 |
| rs11229555 | 58408687 | 1.2147E-08 | -0.0823351 | 11 | 47745 | 0.0144507 | T | G | 0.2515 | 122.1657291 |
| rs11230563 | 60776209 | 1.90296E-08 | -0.0750666 | 11 | 47745 | 0.0133556 | T | C | 0.348 | 122.3973444 |
| rs661054 | 114430410 | 3.18273E-20 | -0.124858 | 11 | 47745 | 0.0135529 | G | A | 0.3408 | 336.776648 |
| rs483905 | 96023427 | 3.15929E-10 | 0.0849927 | 11 | 47745 | 0.0135107 | A | G | 0.289 | 142.154819 |
| rs12318183 | 68503836 | 1.44278E-37 | 0.162178 | 12 | 47745 | 0.0126604 | A | C | 0.3854 | 602.383619 |
| rs76904798 | 40614434 | 2.77984E-09 | 0.104624 | 12 | 47745 | 0.0176013 | T | C | 0.1368 | 123.7439718 |
| rs941823 | 41013977 | 1.39155E-13 | 0.108669 | 13 | 47745 | 0.0146906 | C | T | 0.7509 | 211.8506062 |
| rs55808324 | 88444752 | 1.47092E-09 | 0.127209 | 14 | 47745 | 0.021035 | A | G | 0.09318 | 130.9207442 |
| rs7404095 | 23864590 | 1.52416E-08 | 0.0717686 | 16 | 47745 | 0.0126826 | C | T | 0.5796 | 120.140995 |
| rs11641184 | 11704651 | 4.24043E-10 | 0.0780232 | 16 | 47745 | 0.0124939 | A | C | 0.4762 | 145.4330204 |
| rs9891119 | 40507980 | 1.72346E-11 | -0.0895467 | 17 | 47745 | 0.0133101 | C | A | 0.3536 | 175.6496718 |
| rs17780256 | 70642923 | 6.12774E-13 | -0.115389 | 17 | 47745 | 0.0160316 | C | A | 0.1927 | 198.6040235 |
| rs7240004 | 46395022 | 2.49862E-10 | -0.0823655 | 18 | 47745 | 0.0130179 | G | A | 0.3795 | 153.0290028 |
| rs11083840 | 47119910 | 3.41036E-08 | 0.0691316 | 19 | 47745 | 0.0125262 | G | T | 0.4025 | 110.0008864 |
| rs4812833 | 43068996 | 1.87068E-16 | 0.103346 | 20 | 47745 | 0.0125571 | A | G | 0.5188 | 255.9615712 |
| rs4456788 | 45616324 | 7.06968E-16 | -0.102758 | 21 | 47745 | 0.0127344 | A | G | 0.6106 | 240.9405732 |

**Supplementary Table 4. Characteristics of CD-associated SNPs.**

| SNP | Pos | P | Beta | Chr | Samplesize | SE | EA | NEA | EAF | F |
| --- | --- | --- | --- | --- | --- | --- | --- | --- | --- | --- |
| rs2974935 | 155181843 | 5.80E-10 | 0.0755507 | 1 | 51874 | 0.0121937 | T | G | 0.4948 | 148.4479146 |
| rs10798069 | 186875459 | 4.25E-09 | -0.070387 | 1 | 51874 | 0.0119822 | T | G | 0.4925 | 128.7855366 |
| rs2641348 | 120437884 | 9.65E-10 | -0.121336 | 1 | 51874 | 0.0198422 | G | A | 0.1077 | 147.1972123 |
| rs12411259 | 172866210 | 1.43E-22 | 0.134364 | 1 | 51874 | 0.0137443 | A | G | 0.2401 | 343.9917124 |
| rs3024505 | 206939904 | 3.95E-25 | 0.165311 | 1 | 51874 | 0.0159634 | A | G | 0.1573 | 378.5524883 |
| rs7517847 | 67681669 | 1.38E-159 | -0.335814 | 1 | 51874 | 0.0124758 | G | T | 0.4352 | 3044.48544 |
| rs36016881 | 8051241 | 1.60E-10 | -0.108961 | 1 | 51874 | 0.0170379 | G | A | 0.1754 | 178.7607455 |
| rs1517352 | 191931464 | 1.31E-10 | 0.0800097 | 2 | 51874 | 0.0124517 | C | A | 0.6048 | 159.22382 |
| rs7608910 | 61204856 | 2.95E-23 | 0.120609 | 2 | 51874 | 0.0121405 | G | A | 0.3909 | 361.8224478 |
| rs6738490 | 234161583 | 4.26E-78 | 0.226164 | 2 | 51874 | 0.0120892 | C | T | 0.5274 | 1357.254132 |
| rs35320439 | 242737341 | 9.89E-10 | 0.0840555 | 2 | 51874 | 0.0137546 | C | T | 0.3104 | 157.3728551 |
| rs7438704 | 48363245 | 3.42E-11 | 0.0839019 | 4 | 51874 | 0.0126601 | G | A | 0.6435 | 168.0813934 |
| rs1363907 | 96252803 | 3.89E-16 | 0.102575 | 5 | 51874 | 0.0125984 | A | G | 0.4212 | 267.4832891 |
| rs7711427 | 40414886 | 5.17E-88 | 0.24803 | 5 | 51874 | 0.0124713 | C | A | 0.613 | 1559.580818 |
| rs71624119 | 55440730 | 6.57E-10 | -0.0923221 | 5 | 51874 | 0.0149485 | A | G | 0.2422 | 162.8036001 |
| rs34804116 | 72539850 | 1.27E-13 | -0.0939086 | 5 | 51874 | 0.0126747 | A | C | 0.3867 | 217.8920183 |
| rs9457247 | 167392174 | 2.08E-23 | 0.123663 | 6 | 51874 | 0.0124045 | T | C | 0.5398 | 397.1314946 |
| rs6456426 | 21438889 | 1.37E-16 | -0.0991795 | 6 | 51874 | 0.0119962 | A | C | 0.4984 | 256.3796865 |
| rs11152949 | 106449085 | 2.18E-25 | 0.133849 | 6 | 51874 | 0.0128549 | G | A | 0.3195 | 407.2759956 |
| rs2395022 | 98750379 | 3.13E-10 | -0.177152 | 7 | 51874 | 0.0281553 | C | A | 0.95885 | 128.7812083 |
| rs2538470 | 148220448 | 1.05E-09 | -0.0749643 | 7 | 51874 | 0.0122847 | G | A | 0.6378 | 135.0311602 |
| rs7015630 | 90875918 | 9.00E-10 | -0.0842498 | 8 | 51874 | 0.0137523 | C | T | 0.2657 | 144.0689408 |
| rs6651252 | 129567181 | 3.86E-16 | -0.149029 | 8 | 51874 | 0.0183017 | C | T | 0.13 | 261.9114501 |
| rs7848647 | 117569046 | 1.55E-27 | 0.141308 | 9 | 51874 | 0.0129961 | C | T | 0.6746 | 458.7588048 |
| rs34779708 | 35466185 | 1.90E-27 | 0.134457 | 10 | 51874 | 0.0123872 | G | T | 0.3512 | 430.9114587 |
| rs7085798 | 101288347 | 1.53E-47 | -0.174348 | 10 | 51874 | 0.0120372 | A | C | 0.5176 | 799.5424725 |
| rs2153283 | 59972299 | 2.39E-12 | -0.108804 | 10 | 51874 | 0.0155227 | A | C | 0.217 | 209.5196998 |
| rs2227551 | 75669190 | 4.72E-13 | 0.0994456 | 10 | 51874 | 0.0137489 | T | G | 0.7288 | 203.5791507 |
| rs559928 | 64150370 | 3.75E-10 | 0.099124 | 11 | 51874 | 0.0158237 | C | T | 0.8128 | 155.5645618 |
| rs7969592 | 68579649 | 1.04E-09 | -0.0731898 | 12 | 51874 | 0.011991 | G | A | 0.4746 | 138.9452375 |
| rs76906269 | 40607709 | 1.75E-26 | 0.394341 | 12 | 51874 | 0.0370279 | G | A | 0.01883 | 299.7810014 |
| rs915286 | 40695992 | 2.59E-08 | 0.066645 | 13 | 51874 | 0.0119709 | A | G | 0.5488 | 114.3503935 |
| rs6561151 | 44484706 | 4.68E-25 | 0.147148 | 13 | 51874 | 0.0142318 | A | G | 0.2235 | 392.7959212 |
| rs11159833 | 88476004 | 7.59E-14 | 0.155227 | 14 | 51874 | 0.0207601 | T | C | 0.08681 | 198.9256222 |
| rs1569328 | 75741751 | 6.47E-11 | -0.109215 | 14 | 51874 | 0.0167191 | T | C | 0.1702 | 175.3582704 |
| rs72727394 | 38847022 | 5.28E-12 | 0.103233 | 15 | 51874 | 0.0149661 | T | C | 0.2007 | 177.9691615 |
| rs6500315 | 50508101 | 2.18E-23 | 0.145539 | 16 | 51874 | 0.0146056 | G | A | 0.7753 | 385.6662509 |
| rs7194886 | 50725193 | 1.42E-77 | -0.226986 | 16 | 51874 | 0.0121749 | T | C | 0.4357 | 1348.352663 |
| rs9889296 | 32570547 | 2.96E-25 | -0.143097 | 17 | 51874 | 0.0137815 | A | G | 0.2723 | 424.3876736 |
| rs12949918 | 40526273 | 3.47E-17 | -0.104177 | 17 | 51874 | 0.0123587 | C | T | 0.4185 | 275.4558472 |
| rs3853824 | 54880993 | 1.17E-10 | 0.0813612 | 17 | 51874 | 0.0126283 | C | T | 0.6385 | 158.9996101 |
| rs7236492 | 77220616 | 9.09E-09 | -0.0996611 | 18 | 51874 | 0.017342 | T | C | 0.1537 | 134.3804793 |
| rs6074022 | 44740196 | 2.70E-12 | -0.0963211 | 20 | 51874 | 0.0137745 | T | C | 0.7497 | 181.2462553 |
| rs2284553 | 34776695 | 5.63E-17 | 0.103192 | 21 | 51874 | 0.0123247 | G | A | 0.5904 | 268.5368252 |
| rs8127691 | 45614860 | 4.48E-24 | -0.123357 | 21 | 51874 | 0.0121887 | C | T | 0.6132 | 377.1599045 |
